# Depression and Risk of Dementia: A Burden of Proof Study

**DOI:** 10.1093/geroni/igaf122.2521

**Published:** 2025-12-31

**Authors:** Channa Buxbaum, Natalie Chen, Anh Thy Nguyen, Jamileh Shadid, Kate Gillespie, Damian Santomauro, Liane Ong, Jaimie Steinmetz

**Affiliations:** Institute for Health Metrics and Evaluation, New York, New York, United States; Institute for Health Metrics and Evaluation, Seattle, Washington, United States; Institute for Health Metrics and Evaluation, Seattle, Washington, United States; Queensland Centre for Mental Health Research, Wacol, Queensland, Australia; IHME Client Services, Seattle, Washington, United States; Queensland Centre for Mental Health Research, Wacol, Queensland, Australia; Institute for Health Metrics and Evaluation, Seattle, Washington, United States; Institute for Health Metrics and Evaluation, Seattle, Washington, United States

## Abstract

Depression is widely considered a risk factor for dementia, yet its precise relationship remains difficult to quantify due to substantial variability in measurement approaches across studies. In the Global Burden of Disease (GBD) study, we estimate that the number of people living with a depressive disorder globally has more than doubled from 1990 to 2024, now affecting approximately 307 million people. Given its growing burden, clarifying its role in dementia risk is imperative. To address this, we applied the Burden of Proof framework, developed for the GBD study, to adjust for systematic biases and account for heterogeneity. We conducted a systematic review and meta-analysis of longitudinal studies in PubMed, Web of Science, and PsycInfo published between 2012–2024, building on a prior meta-analysis covering 1980–2012. Of 9,625 references, 41 met eligibility criteria, with 18 included after deduplication and removal of registry-based studies. Our initial pooled analysis found a moderate-to-strong association between depression and dementia (RR = 1.54, 95% UI: 1.32–1.79). However, when restricting to nine studies with gold-standard depression measures—excluding studies relying on symptom scales, self-report, or potentially non-representative populations—the estimated association became more uncertain (RR = 1.26, 95% UI: 0.86–1.87). These results underscore how inconsistencies in depression measurement can alter the perceived strength of the depression-dementia relationship. While depression remains a plausible contributor to dementia risk, these findings emphasize the importance of rigorous, standardized assessment in epidemiological research. As the burden of depression rises globally, ensuring methodological rigor in future studies will be essential for accurately informing dementia prevention strategies.

